# Coronavirus Disease 2019 and Acute Hemorrhagic Edema of Infancy

**DOI:** 10.1155/2022/7610402

**Published:** 2022-02-03

**Authors:** Mohsen Jari

**Affiliations:** Department of Pediatric Rheumatology, Imam HosseinChildren,s Hospital, Isfahan University of Medical Sciences, Isfahan, Iran

## Abstract

**Background:**

Acute hemorrhagic edema of infancy (AHEI) is a small-vessel leukocytoclastic vasculitis, presented with low-grade fever and edema in the face and upper and lower limbs, as well as purpuric/ecchymotic lesions in these regions. AHEI was also reported after viral infections, including herpes simplex virus, rotavirus, and adenovirus. *Case Presentation*. Herein, we reported a case of a 20-month-old boy presented with low-grade fever. Additionally, mild cough and progressive purpuric/ecchymotic lesions were observed in some independent regions and extremity swelling. Laboratory testing showed leukopenia, lymphopenia, and the elevation of both C-reactive protein (CRP) and erythrocyte sedimentation rate (ESR). Thereafter, the result of the reverse-transcriptase polymerase chain reaction (RT-PCR) test on the obtained specimen samples, including nasopharyngeal swab for COVID-19, was positive. The patient was treated with supportive care, and two weeks later, the serology test for COVID-19 resulted positive.

**Conclusion:**

We should think about children infected with COVID-19, particularly those with skin manifestations.

## 1. Introduction

Acute hemorrhagic edema of infancy (AHEI) is an immune complex-mediated leukocytoclastic vasculitis, known as a rare disease in children [1]. Age at the time of the onset of this disease is usually between 4 and 24 months. Clinical findings such as low-grade fever, edema of the face and upper and lower limbs, generalized purpura, or ecchymosis rapidly develop over 24–48 hours. Upper respiratory tract infection, gastroenteritis, or vaccination has also been reported to cause this disease [2, 3]. To the best of our knowledge, this is the first report on the association between coronavirus disease 2019 (COVID-19) and AHEI.

## 2. Case Presentation

A 20-month-old boy was admitted to the pediatric rheumatology clinic of Imam Hossein Children's Hospital, Isfahan University of Medical Sciences, due to fever, mild cough, progressive purpuric/ecchymotic lesions, and extreme swelling in the past 2 days.

The rash initially began on his legs and then rapidly diffused to his forearms, face, and ears.

**Figure 1:**
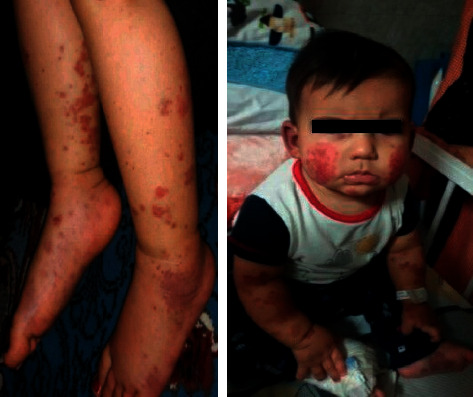
Extremity swelling and purpuric/ecchymotic lesions on the limbs and face.

In physical examination, he was a happy and healthy baby. Besides rash, swelling of his hands and feet and nonexudative bilateral conjunctivitis were visible as well (Figure 1).

He had mild cough for a 5-day duration. Of note, his father was hospitalized due to COVID-19 10 days ago. Review of systems was negative regarding any other problem.

Vital signs were notable for a fever of 38.2°C along with normal respiratory rates and blood pressure.

Laboratory testing demonstrated leukopenia (white blood cell count: 3800/mm^3^) and lymphopenia (lymphocyte: 16%). Moreover, the elevation was observed in C-reactive protein (CRP: 47 mg/L) and erythrocyte sedimentation rates (ESR: 19 mm/hr). In addition, serum electrolytes, renal function tests, urinalysis, prothrombin time, and the activated partial thromboplastin time were normal. A reverse-transcriptase polymerase chain reaction (RT-PCR) test was performed on the obtained specimen samples, including nasopharyngeal swab for COVID-19, which resulted positive, while the result of the serology test (Ig M and Ig G) for COVID-19 was negative. Additionally, the chest X-ray graph was normal.

The patient was recognized as a case of AHEI triggered by COVID-19. Thereafter, the patient was isolated at home and treated only with supportive care (good nutrition and observation). He had resolution of all symptoms within 5 days. Subsequently, the isolation at home was continued. Two weeks later, a RT-PCR test was performed on the specimen samples, including a nasopharyngeal swab test for COVID-19, which was negative, while the serology test (Ig M) for COVID-19 was positive.

## 3. Discussion

AHEI was reported after upper respiratory tract infection and viral infections, including herpes simplex virus, rotavirus, and adenovirus. In this regard, vaccinations and antibiotics have been recognized as triggers. AHEI is a leukocytoclastic vasculitis in infants and toddlers aged less than 2 years. This disease is characterized by a purpuric/ecchymotic rash and edema over the limbs, ears, and face. IgA deposits are seen in 10% to 35% of biopsy specimens, so this is a disagreement that it is a mild form of HSP or it is a distinct disease [3, 4]. Usually, AHEI is a self-limited disease, and more than 90% of infected patients completely recover from the disease only with a supportive care within 1–3 weeks after the symptoms' presentation. Of note, systemic involvement, including abdominal pain, intussusception, arthralgia, testicular torsion, and glomerulonephritis, occurred in fewer than 10% of patients diagnosed with AHEI [4, 5].

Diagnosis of AHEI is clinical. Leukocytosis and thrombocytosis and the elevation of C-reactive protein (CRP) and erythrocyte sedimentation rate (ESR) may occur as well. The treatment of AHEI is known as a supportive care, while in the systemic involvement, using corticosteroids remains controversial yet. Corticosteroids can only be considered in a severe systemic involvement and in complications resulted from AHEI3 [3, 4].

The use of both antihistamines and steroids is controversial. Systemic corticosteroids may be used under sever conditions or systemic involvement such as abdominal pain, intussusception, nephritis, scrotal pain, and testicular torsion. Monitoring patients with a rare systemic involvement such as renal involvement is recommended [4, 6].

Currently, the coronavirus disease 2019 (COVID-19) is a pandemic worldwide. COVID-19 increased mortality and morbidity rates in rheumatologic patients due to the underlying immune dysfunction and the treatment with immunosuppressive agents [7].

There are few reports demonstrating that COVID-19 is associated with maculopapular, purpuric, and acroischemic skin lesions. In this regard, few reports suggested that COVID-19 triggers the secretion of some inflammatory products and induces generalized vasculitis. It may be considered as the etiology of these vasculitis-like lesions due to causing endothelial damage [8, 9].

Although no test is suggested in this regard, especially in healthy outpatients, the Italian Rhinologic Society recommended “against the performance of rhinomanometry, acoustic rhinometry, and olfactometry, the self-rating nasal symptoms grading using the Visual Analog Scale or validated questionnaires is the best way to approach these patients. The skin-prick test could also be performed as an alternative. Post-COVID-19 patients could be rather tested clinically and through functional tests, and the Italian Rhinologic Society recommended the evaluation of these patients after 3 consecutive negative swabs (the last one in the previous 48 h)” [10].

Clinical manifestations of coronavirus disease 2019 (COVID-19) are rare or ambiguous among children and adolescents. There are some reports on COVID-19 infection in these age groups, presenting with some types of skin lesions such as erythematous rashes, urticaria, erythematous violaceous patches, purpuric lesions, blisters and ulceronecrotic lesions, and chicken pox-like vesicles.

During the current COVID-19 pandemic, we should think about this infection in all children and adolescents with some skin manifestations [11–13].

## 4. Conclusions

Some viruses were reported to be associated with AHEI, and our case demonstrated that COVID-19 can precede AHEI as well.

## Abbreviations

AHEI: Acute hemorrhagic edema of infancy
COVID-19: Coronavirus disease 2019
CRP: C-reactive protein
ESR: Erythrocyte sedimentation rate
RT-PCR: Reverse-transcriptase polymerase chain reaction.
